# Image‐guided volumetric‐modulated arc therapy of total body irradiation: An efficient workflow from simulation to delivery

**DOI:** 10.1002/acm2.13412

**Published:** 2021-09-04

**Authors:** Bingqi Guo, Cherian Sheen, Erin Murphy, Anthony Magnelli, Lan Lu, YoungBin Cho, Peng Qi, Navneet S Majhail, Ping Xia

**Affiliations:** ^1^ Department of Radiation Oncology Taussig Cancer Institute Cleveland Clinic Cleveland Ohio USA; ^2^ Department of Hematology Oncology Taussig Cancer Institute Cleveland Clinic Cleveland Ohio USA

**Keywords:** IGRT, sim‐to‐treat, TBI, VMAT

## Abstract

**Introduction:**

Using multi‐isocenter volumetric‐modulated arc therapy (VMAT) for total body irradiation (TBI) may improve dose uniformity and vulnerable tissue protection compared with classical whole‐body field technique. Two drawbacks limit its application: (1) VMAT‐TBI planning is time consuming; (2) VMAT‐TBI plans are sensitive to patient positioning uncertainties due to beam matching. This study presents a robust planning technique with image‐guided delivery to improve dose delivery accuracy. In addition, a streamlined sim‐to‐treat workflow with automatic scripts is proposed to reduce planning time.

**Materials:**

Twenty‐five patients were included in this study. Patients were scanned in supine head‐first and feet‐first directions. An automatic workflow was used to (1) create a whole‐body CT by registering two CT scans, (2) contour lungs, kidneys, and planning target volume (PTV), (3) divide PTV into multiple sub‐targets for planning, and (4) place isocenters. Treatment planning included feathered AP/PA beams for legs/feet and VMAT for the body. VMAT‐TBI was evaluated for plan quality, planning/delivery time, and setup accuracy using image guidance.

**Results:**

VMAT‐TBI planning time can be reduced to a day with automatic scripts. Treatment time took around an hour per fraction. VMAT‐TBI improved dose coverage (PTV V100 increased from 76.8 ± 10.5 to 88.5 ± 2.6; *p* < 0.001) and reduced lung dose (lung mean dose reduced from 10.8 ± 0.7 Gy to 9.4 ± 0.8 Gy, *p* < 0.001) compared with classic AP/PA technique.

**Conclusion:**

A VMAT‐TBI sim‐to‐treat workflow with robust planning and image‐guided delivery was proposed. VMAT‐TBI improved the plan quality compared with classical whole‐body field techniques.

## 1 INTRODUCTION

Total body irradiation (TBI) is an integral part of conditioning regimens for patients undergoing hematopoietic stem cell transplantation.^1^, ^2^, ^3^ Traditionally, TBI is treated with large opposed whole‐body fields (e.g., AP/PA) at extended distances.^4^ Lung blocks and kidney blocks are often used to reduce lung and kidney doses. A major limitation of the classic techniques is that recent technological advances in image‐guided organ sparing intensity‐modulated radiation therapy have not been applied to TBI treatments. Multi‐isocenter, volumetric‐modulated arc therapy (VMAT) for TBI has been proposed recently.^5^, ^6^, ^7^, ^8^, ^9^ VMAT‐TBI provides better control of dose distribution to the whole body and improves the relative sparing of vulnerable structures.

Planning for VMAT‐TBI is a complex, time‐consuming process that usually takes days.^6^ Besides difficulties in planning, VMAT‐TBI delivery is sensitive to positioning errors due to intensity modulation and dose matching for multiple isocenters.^10^ To reduce planning time and complexity, this study presents a streamlined sim‐to‐treat VMAT‐TBI workflow, with automatic scripts. To alleviate positioning errors, a robust planning technique was developed for VMAT‐TBI and a workflow for image‐guided radiation therapy (IGRT) delivery of VMAT‐TBI was implemented.

## 2 MATERIAL AND METHODS

### 2.1 Patients

Twenty‐five patients were included in this study, of which 21 were adult patients and four were pediatric patients. Two myeloablative TBI prescriptions were used: eight patients were treated to 12 Gy in eight fractions and 17 patients were treated to 13.2 Gy in eight fractions. All patients were treated BID, 6 h apart. Table 1 lists patient characteristics.

**TABLE 1:** Patient characteristics

Number of patients	25
Age	9–59
Gender	M(18), F(7)
Diagnosis	ALL(19), AML(3), others(3)
Prescription	165 cGy × 8 (17), 150 cGy × 8 (8)
Height (cm)	139–193
Weight (kg)	25.7–126.1
Umbilicus separation (cm)	14.3–33.0

### 2.2 CT simulation

**FIGURE 1:**
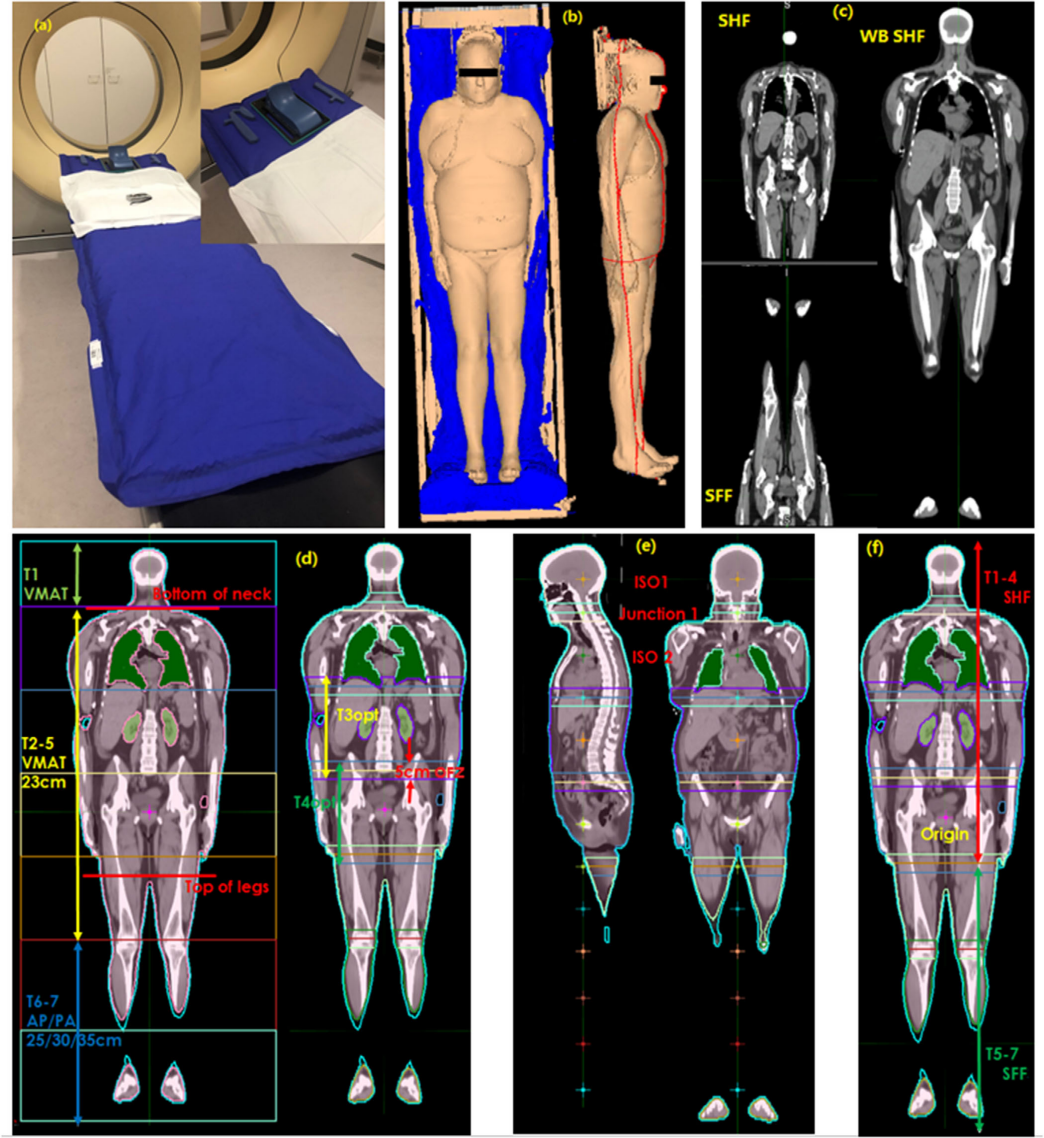
CT simulation, volumes of interest, and points of interest. (a) Immobilization devices; (b) patient positioning; (c) supine head first, supine feet first, and whole‐body CT scans; (d) sub‐target volumes and optimization feathering zone (OFZ); (e) isocenters and junction points; and (f) head‐first and feet‐first sub‐targets

Figures 1(a)–1(c) show the equipment and process for CT simulation using a Philips Big bore CT (Philips, Amsterdam, Netherlands) The immobilization devices included a full‐body vacuum bag with a head plate to hold an open face mask. Patients were positioned supine with arms tightly adducted to the body sides and with legs straight. Two CT scans were acquired: a supine head‐first (SHF) scan from above the head to below the pelvis and a supine feet‐first (SFF) scan from below the feet to above the pelvis. Both scans were acquired with 5 mm slice thickness. A MIM (version 7.0.7; MIM Inc., Cleveland, OH) workflow was developed to rigidly register the SHF scan and the SFF scan in the pelvis region and then create a whole‐body CT. A reference origin point was marked in the center of the pelvis.

### 2.3 Volumes of interest and points of interest

Figures 1(d)–1(f) illustrate the volume of interests and points of interest (POIs) for VMAT‐TBI planning. Two critical structures, lungs and kidneys, were contoured using atlas‐based auto segmentation. The planning target volume (PTV) was defined as the whole body, contracted 5 mm (3 mm in each direction) in from the skin, and excluding the lungs and kidneys. The whole‐body PTV was divided into multiple sub‐targets for planning. Legs and feet (<= 35 cm in width) were planned with AP/PA beams and the rest part of the body was planned with VMAT beams. A MIM workflow was developed to automatically divide PTV into VMAT and AP/PA sub‐targets. The first VMAT sub‐target extended from the top of the head to the bottom of the neck. Subsequent VMAT sub‐targets, each measuring 23 cm in the superior–inferior direction, extended from the bottom of the neck to below the top of the legs. AP/PA sub‐targets, which could be 25, 30, or 35 cm in length, covered the rest of the body including legs and feet. Each of the sub‐targets was then extended 2.5 cm superiorly and inferiorly to create 5 cm optimization feathering zones (OFZs), as shown in Figure 1(d). For an adult patient, there were usually seven or eight sub‐targets, with the last two or three being AP/PA sub‐targets and the rest being VMAT sub‐targets.

The MIM workflow automatically placed an isocenter at the center of each sub‐target and a junction point at the center of each junction region. All the points had the same lateral and vertical coordinates, varying only in the longitudinal direction.

The optimal division of VMAT and AP/PA sub‐target lengths was a result of the planning techniques, which is explained in Section 2.4.4.

### 2.4 Robust VMAT‐TBI treatment planning

**FIGURE 2:**
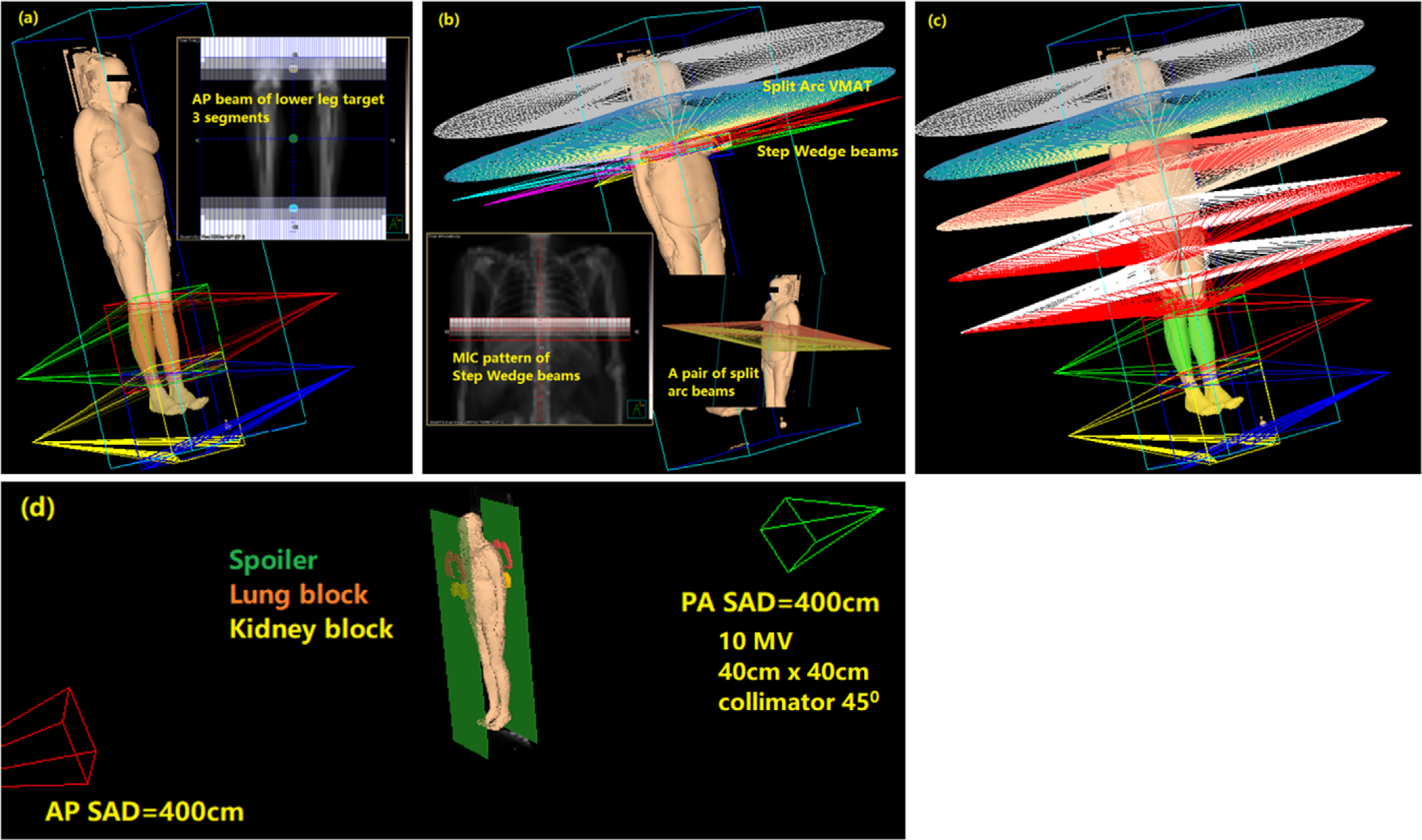
Illustration of robust VMAT‐TBI treatment planning and comparison with classic AP/PA technique. (a) AP/PA beams with beam feathering for leg sub‐targets; (b) step wedge beams to create a base dose for optimization, and split arc VMAT beams; (c) whole‐body VMAT plan; (d) recreate the whole‐body AP/PA plan for comparison

In this study, all patients were planned in Pinnacle3 (Philips Inc., Fitchburg, WI) treatment planning system, version 16.2 with Varian Truebeam linear accelerator (Varian Inc., Palo Alto, CA). The whole‐body CT was used to create a whole‐body VMAT plan, which included feathered AP/PA technique for AP/PA sub‐targets and robust VMAT planning technique for VMAT sub‐targets, as illustrated in Figures 2(a)–2(c). In‐house Pinnacle scripts were developed to facilitate beam placement, optimization, and exporting the whole‐body plan to head‐first and feet‐first directions for delivery. Five millimeters dose grid was used.

#### 2.4.1 AP/PA technique with beam feathering

Each AP/PA sub‐target was planned with a pair of 6MV AP/PA beams with the collimator angle set at 90°. Each beam had three segments. The segments were rectangular fields with Y‐jaws (lateral) set at maximum and varying MLC positions, as shown in Figure 2(a). These segments with variable field sizes created dose feathering to improve the dose uniformity in junction regions and reduce the sensitivity of the plan to set up errors or patient movement. Beam segment weights were optimized to deliver a uniform dose to the AP/PA sub‐targets. The last pair of AP/PA beams had the inferior jaw and MLC set to maximum size (20 cm) to ensure adequate field margin beyond the feet.

#### 2.4.2 Using step wedge beams to improve robustness in planning

To create a gradual dose fall‐off at the junction regions when optimizing each VMAT sub‐target, we added a wedged dose distribution at the junction region and included the wedged dose as a base dose in optimization. Nine equally weighted step‐and‐shoot beams with gantry angles 40° apart were utilized. Each beam had 5 equally weighted segments forming a 5 cm wide step wedge intensity pattern, as shown in Figure 2(b). The step‐wedge beams created a 5 cm wide, wedge‐shaped dose distribution in the superior–inferior direction. Optimization with the wedged dose as base dose forced the VMAT beams to deliver a dose distribution that gradually falls off in the junction region. The step‐wedge beams were used to assist in planning only. They were not included in the final VMAT TBI plan for delivery.

#### 2.4.3 VMAT planning

The VMAT sub‐targets were planned sequentially from the head to pelvis. Full arcs with collimator angle of 90° were used for all VMAT beams. Except for the head sub‐target, the split arc technique (as shown in Figure 2(b)) was applied for all VMAT sub‐targets to reduce the MLC travel distance. Usually, two pairs of split arcs were needed for the lung sub‐target, whereas one pair of split arc was sufficient for other VMAT sub‐targets. Optimization goals were to achieve uniform dose within the optimization sub‐targets (sub‐targets plus OFZs) and relative sparing of vulnerable structures.

After all, VMAT sub‐targets were optimized, a segment weight optimization of all VMAT beams was performed to further improve dose uniformity. Figure 2(c) shows all the beams for a full body VMAT TBI plan.

The optimization goals included PTV coverage, hotpots, lung, and kidney sparing^2^:

- PTV V100 >= 90%, PTV V95 >= 95%, PTV V110 <= 20%
- Whole_Lung Dmean <= 10 Gy (adult patients) or <= 8 Gy (pediatric patients)
- Whole_Kidney Dmean <= 6 Gy

6 MV was preferred to increase skin dose although 10 MV may be used for large‐sized patients. For VMAT beams at the lung isocenter, 200 MU/min beam monitor unit (MU) rate was used, whereas for all other beams, a MU rate of 600 MU/min was applied.

#### 2.4.4 Optimal sub‐target lengths

The choice of VMAT and AP/PA sub‐target lengths in auto segmentation depended on the treatment planning system and treatment machine. For VMAT sub‐targets, we found that plan quality was best when field width in the superior–inferior direction was not more than the MLC travel limit of 14.5 cm. With the use of split arcs, we extended the field size to 14.5 × 2 = 29 cm. A 28 cm length limit was applied to the optimization sub‐targets (sub‐targets plus 5 cm target feathering zone) to give the field a 5 mm margin on each side. Therefore, the length limit for the VMAT sub‐targets was set at 23 cm. For most adult patients, 23 cm also conveniently separated the body into lung, abdomen, and pelvis regions. For pediatric patients, the workflow was edited to shorten the lengths of sub‐targets in lung and abdomen so that the junction region was placed between the lungs and kidneys. For large‐sized patients, the length was reduced by 1 or 2 cm to allow adequate coverage of the arms.

All AP/PA sub‐targets were the same length to match beam divergence at the junction, but their length can be 25, 30, or 35 cm, depending on the remaining length of the body after all the VMAT sub‐targets were defined. The goal was to cover the remaining PTV with as few isocenters as possible, ensure an adequate field margin beyond feet (>= 5 cm) while reducing the isocenter distances. The MIM workflow used a look‐up table to determine the AP/PA sub‐target length.

### 2.5 Recreate the AP/PA whole‐body fields for plan comparison

For plan comparison, we recreated a classic AP/PA plan on the whole‐body CT images following our institutional protocol before VMAT‐TBI implementation, as shown in Figure 2(d). The maximum thickness of the patient's body was measured from the CT images for beam MU calculation. Both AP and PA beams had 10 MV energy, 40 cm by 40 cm field size with collimator angle at 45°, and a source to axis distance of 400 cm. A 1 cm thick spoiler made of Lucite (density 1.18 g/cm^3^) was placed in the beam near the patient to increase skin dose. The lung and kidney blocks made of cerrobend (density 9.4 g/cm^3^) were created from structure contours. Lung blocks contracted from the lung‐heart outline in the beam's eye view by 5 mm. The lung block thickness was calculated to deliver 10 and 8 Gy at the mid‐lung position for adult and pediatric patients, respectively. The kidney blocks had the shape of kidneys in the beam's eye view and a thickness of 4 cm. The spoiler, lung blocks, and kidney blocks were mimicked by density override in the TPS. The dose was calculated in the TPS and exported for comparison.

### 2.6 Convert whole‐body plan into head and feet‐first plans for delivery

For treatment delivery, the whole‐body VMAT‐TBI plan had to be separated into an SHF plan and an SFF plan. The SHF plan contained the copied beams with isocenters superior to the origin point from the whole‐body plan and set for delivery in the SHF direction. To create the SFF plan, we used a “rotate series” tool in MIM to rotate the whole‐body CT images along with the ROIs and the POIs from SHF to SFF. An in‐house Pinnacle script was utilized to invert the beams from the whole‐body plan to the SFF position. The script rotated the collimator angle by 180° and mirrored the gantry angle in the left‐right direction for each beam segment. The SFF plan was calculated to make sure beam MUs and dose distribution stayed the same as the whole‐body plan. Beams with isocenters inferior to the origin point were exported from the SFF plan to be delivered in the SFF direction.

### 2.7 Image‐guided VMAT‐TBI delivery

**FIGURE 3:**
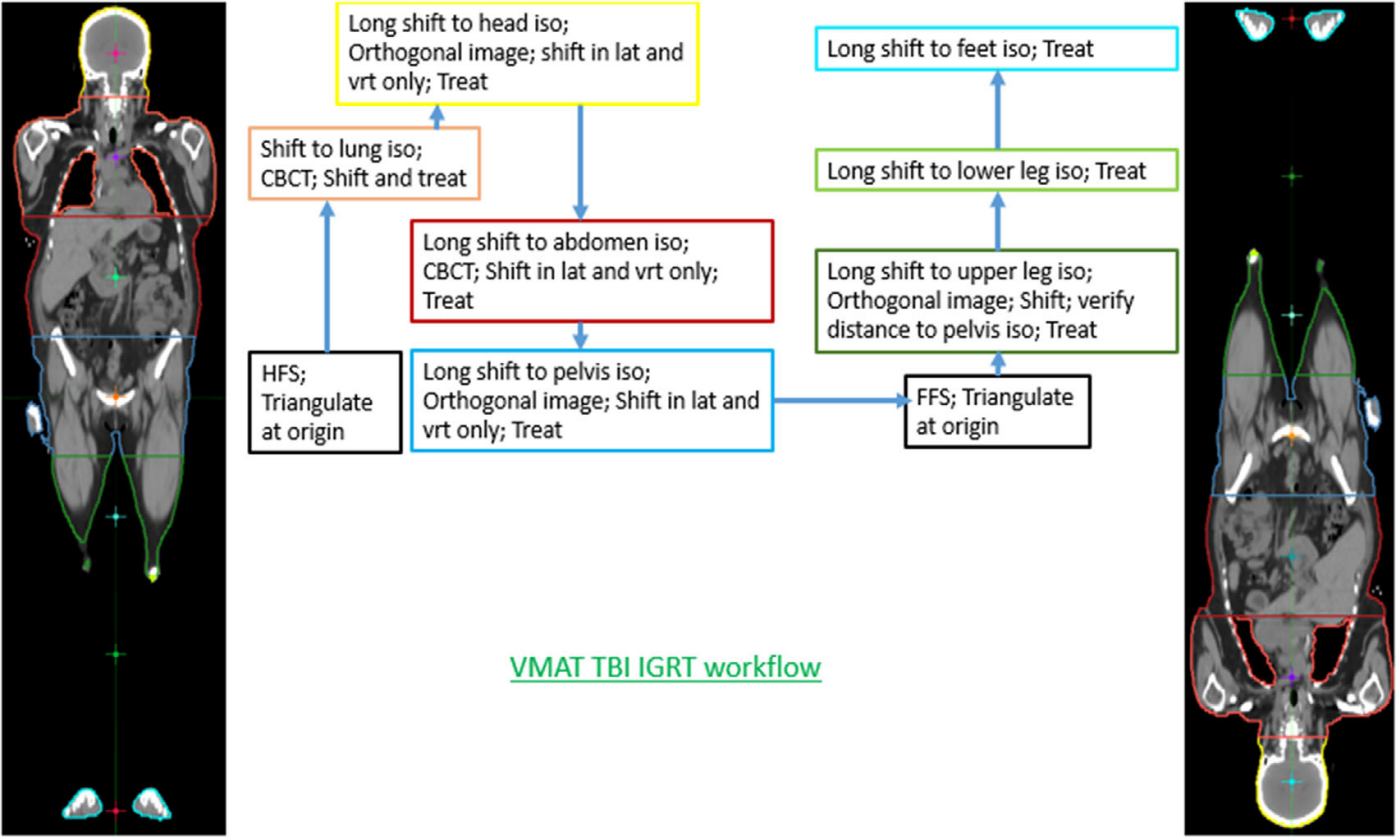
VMAT‐TBI image‐guided delivery workflow

To balance the setup accuracy and delivery time, both CBCT‐based and kilo‐voltage orthogonal imaging‐based setups were used. Figure 3 shows the workflow of image‐guided delivery for VMAT‐TBI. CBCT was used for visualization of soft tissue for lung and abdominal alignment. Orthogonal imaging was used at other isocenters to align the bony anatomy. To ensure dose matching between sub‐targets, table longitudinal shifts between isocenters were calculated from the plan and were not permitted to alter during treatment. Only vertical and lateral shifts were allowed from imaging.

## 3 RESULTS

### 3.1 Planning and delivery time of VMAT‐TBI

The entire process that included the creation of whole‐body CT, delineation of the PTV, division of sub‐targets, and placement of isocenters and junction points took around 5 min using an in‐house developed automatic MIM workflow.

The planning time for multi‐isocenter VMAT‐TBI was approximately 8–12 h with a set of in‐house developed Pinnacle planning scripts. Most time was spent on sequentially optimizing each sub‐target.

The beam on time of VMAT‐TBI delivery was comparable with the classic AP/PA technique. For an example patient (patient #4), the total treatment time, from getting the patient on the treatment table to complete treatment delivery, measured 1 h and 6 min on average for the eight treatment fractions.

### 3.2 Plan quality comparison between AP/PA and VMAT techniques

**FIGURE 4:**
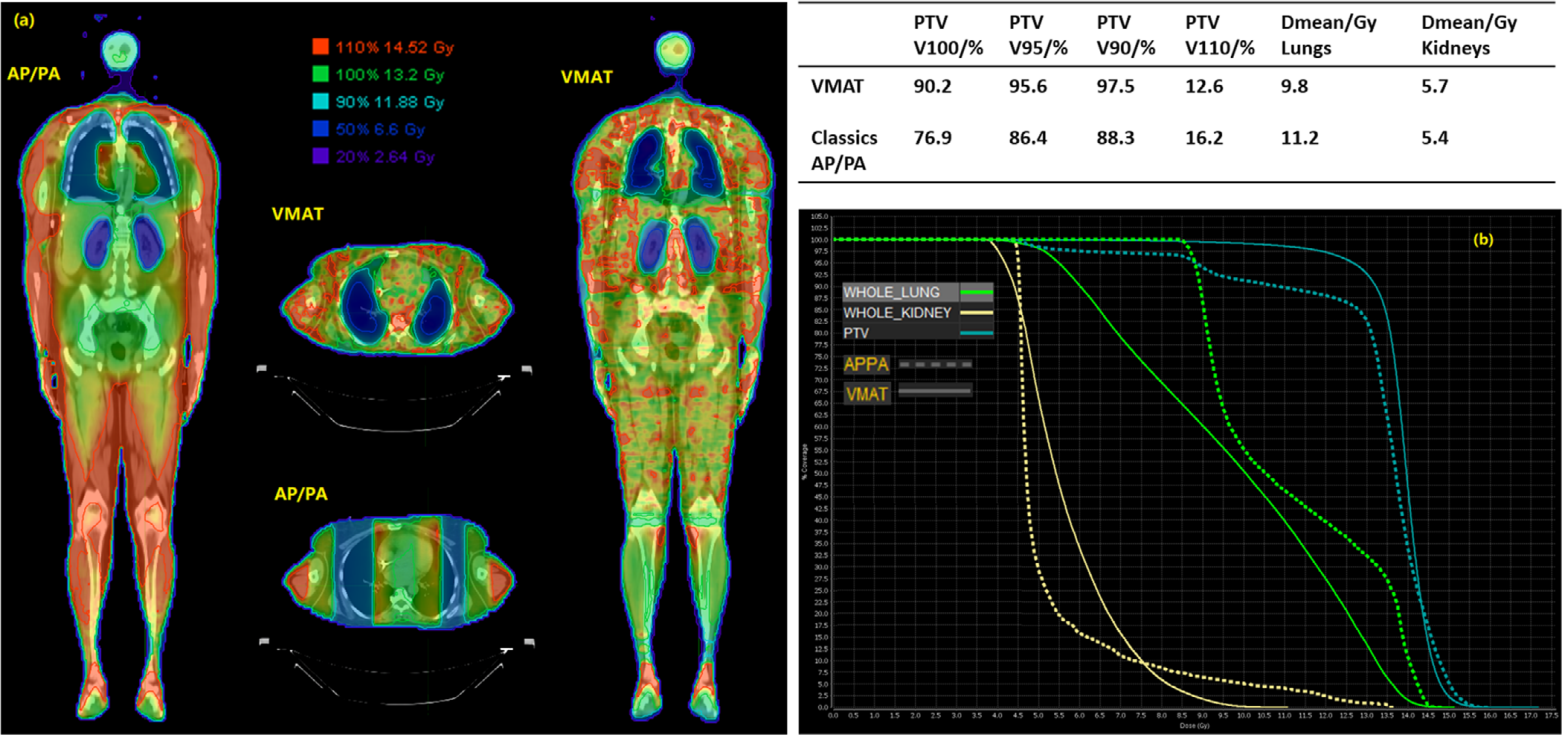
(a) Isodose and (b) DVH comparison of the VMAT technique with classic AP/PA technique for an example patient

Figure 4 compares the dose distribution, dose–volume histogram (DVH), and plan objectives between classic AP/PA whole‐body fields and VMAT‐TBI for the example patient. VMAT‐TBI improved the PTV coverage, reduced the hotspots, and reduced the lung dose compared with the AP/PA technique. The mean dose to the kidney was 6 Gy, meeting the planning goal.

Table 2 compares the plan objectives between VMAT‐TBI and classic AP/PA techniques for 25 patients. Paired, two‐sided *t*‐test was used for statistical analysis with significance defined as *p* <= 0.05. VMAT improved the PTV coverage and reduced the lung dose. For hotspot and kidney dose, the differences were not statistically significant.

**TABLE 2:** Comparison of VMAT‐TBI with classic AP/PA technique for 25 patients

	VMAT	AP/PA	*p* (Paired, two‐sided *t*‐test)
PTV V100 (%)	88.5 ± 2.6	76.8 ± 10.5	<0.001
PTV V95 (%)	94.8 ± 1.6	87.7 ± 3.1	<0.001
PTV V90 (%)	96.9 ± 1.2	90.0 ± 1.8	<0.001
PTV V110 (%)	24.4 ± 13.8	21.5 ± 13.8	0.455
Dmean (Gy Whole_Lung)	9.4 ± 0.8	10.8 ± 0.7	<0.001
Dmean (Gy Whole_Kidney)	6.1 ± 0.8	5.8 ± 0.9	0.09

### 3.3 Lung dose rate of VMAT‐TBI

A major concern of TBI is lung toxicity.^11^ A lower dose rate has been shown to reduce lung toxicity.^12^, ^13^ In classic AP/PA treatments, open beams are delivered at a low MU rate to reduce the lung dose rate to no more than 15 cGy/min. For the VMAT‐TBI technique, depending on the planning system and machine, maintaining a low dose rate (<15 cGy/min) at the lungs may not always be achievable.

In this study, the mean lung dose rate was 15.7 ± 3.1 cGy/min when using a beam MU rate of 200 MU/min. Reducing the beam, MU rate can reduce the lung dose rate at the cost of a longer delivery time.

### 3.4 Image‐guided VMAT‐TBI delivery

**FIGURE 5:**
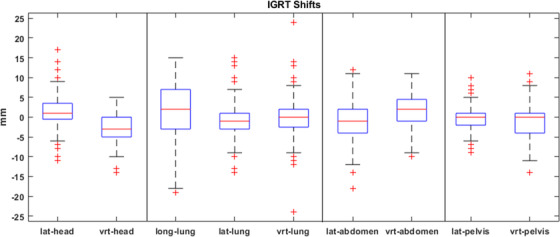
Shifts at head, lung, abdomen, and pelvis isocenters from daily imaging

The patient position shifts based on CBCT or orthogonal images at the head, lung, abdomen, and pelvis isocenters for all 200 treatment fractions of 25 patients were shown in Figure 5. In 38%, 62%, 39%, and 33% of all treatment fractions, shifts at the head, lung, abdomen, and pelvis isocenters were more than 5 mm in any direction, respectively. In 90% of all treatment fractions, imaging shifts were more than 5 mm at one or more isocenters, which proved the importance of image guidance in VMAT‐TBI delivery.

## 4 DISCUSSION

The advantages of VMAT‐TBI are multi‐fold. First, it can be delivered in a conventional linear accelerator room, whereas traditional whole‐body field techniques require a special TBI treatment vault to position patients at extended distances. Second, VMAT‐TBI is more patient friendly. Traditional AP/PA techniques often require the patients in a standing position during the entire delivery, which can be difficult for many TBI patients. Third, the possibility of imaging at each isocenter increases the dose delivery accuracy of VMAT‐TBI. Finally, VMAT‐TBI improves plan quality compared with the traditional AP/PA technique: VMAT‐TBI delivers a more uniform dose to the body and reduces lung dose.

The main drawback of VMAT‐TBI is the extended planning time. In the study by Springer et al.,^6^ contouring of VMAT‐TBI took 5–6 h, and optimization and dose calculation took 25–30 h. Auto‐segmentation and auto‐planning scripts may be used to reduce planning time. In this study, MIM auto image processing and segmentation workflows reduced the contouring time for VMAT TBI to a few minutes. With the assistance of Pinnacle planning scripts, the planning time can be reduced to a day.

The delivery time of VMAT TBI was also slightly longer than that of conventional AP/PA treatments. One hour treatment slot is needed to schedule a VMAT TBI treatment fraction. The majority of time was spent on setting the patient up in both head‐first and feet‐first directions. A dedicated rotational tabletop may reduce setup time.^7^ Daily image guidance also added to the delivery time, but it was necessary to ensure delivery accuracy.

Intensity‐modulated radiation therapy for whole‐body irradiation was first implemented on Tomotherapy (Accuray Inc., Sunnyvale, CA) units.^14^, ^15^, ^16^, ^17^ Tomotherapy‐TBI treats the whole body in two sessions: a SHF session and a SFF session. Although both Tomotherapy and VMAT are able to improve dose uniformity and lung sparing, VMAT‐TBI has a few advantages compared with Tomotherapy: VMAT is more widely available; VMAT‐TBI allows imaging at every isocenter, whereas Tomotherapy TBI only allows imaging for each session; VMAT‐TBI allows to control dose rate of the lungs by changing the beam MU rate at the lung isocenter.

## 5 CONCLUSION

A robust treatment planning technique and an image‐guided delivery workflow were developed for VMAT‐TBI. Planning efficiency was improved using automation with scripts. Compared with the classic AP/PA whole‐body fields, VMAT‐TBI improved the target dose coverage and lung sparing. IGRT is important to ensure the delivery accuracy of VMAT‐TBI.

## CONFLICT OF INTEREST

No conflict of interest related to this study.

## AUTHOR CONTRIBUTION

Bingqi Guo and Ping Xia contributed to all aspects of the research, including study design, data acquisition, data analysis, drafting the manuscript, and manuscript revisions. Cherian Sheen, Erin Murphy, and Navneet S Majhail contributed to study design, data acquisition, and manuscript revisions. Anthony Magnelli, Lan Lu, Youngbin Cho, and Peng Qi contributed to data acquisition, data analysis, and manuscript revisions.
